# Leptin receptor deficiency induces early, transient and hyperglycaemia-independent blood-brain barrier dysfunction

**DOI:** 10.1038/s41598-019-39230-1

**Published:** 2019-02-27

**Authors:** Noa Corem, Shira Anzi, Sivan Gelb, Ayal Ben-Zvi

**Affiliations:** Department of Developmental Biology and Cancer Research, The Institute for Medical Research Israel-Canada, Faculty of Medicine, Hebrew University of Jerusalem, Jerusalem, 91120 Israel

## Abstract

Diabetes mellitus (DM) significantly increases susceptibility to central nervous system (CNS) pathologies, including stroke, vascular dementia, cognitive deficits and Alzheimer’s disease. Previous studies (mostly using the streptozotocin model) suggested that blood-brain barrier (BBB) disruption is involved in these conditions. Here, we examined the integrity of brain capillaries and BBB permeability in Lepr^db/db^ obesity-related diabetic mice. Surprisingly, significant BBB leakage was observed only in young mice at the pre-hyperglycemic stage. Thorough examination of barrier permeability at later diabetic stages showed no evidence for significant BBB leakage during the hyperglycemic state. Electron microscopy imaging of mice with short-term hyperglycaemia supported normal BBB permeability but indicated other stress-related changes in capillary ultrastructure, such as mitochondrial degeneration. Based on our study with this mouse genetic model of obesity-related DM, we suggest that previously reported hyperglycaemia-induced BBB leakage is most likely not the underlying mechanism of DM-related CNS pathologies. Finally we propose that BBB hyper-permeability might be an early and transient phenomenon while stress-related endothelial pathologies do correlate with a short-term diabetic state.

## Introduction

The clinical features and biological mechanisms underlying the microvascular complications of diabetes mellitus (DM) have been extensively studied, in particular insults to three different capillary beds resulting in retinal microvascular dysfunction (DM retinopathy)^1^, renal microvascular dysfunction (DM nephropathy)^2,3^ and microvascular dysfunction of the capillary bed that supplies peripheral nerves (DM neuropathy)^4,5^. In addition to target organs (retina, kidney and peripheral nerves), DM-related insults to the central nervous system (CNS) have been reported in both type 1 and type 2 diabetic patients. It is widely accepted that DM significantly increases susceptibility to multiple CNS pathologies, including stroke, vascular dementia, ventricular hypertrophy, lacunar infarcts, haemorrhage, and seizure disorders^6^. Another DM-related CNS condition is cognitive deficits: individuals with type 2 diabetes have at least a two-fold higher risk for developing significant reduction in cognitive function^6–9^. A longitudinal study showed that elderly subjects with type 2 diabetes had a greater risk of developing amnestic mild cognitive impairment, the transitional state between normal cognitive functioning and Alzheimer’s disease, compared to elderly individuals without diabetes^10,11^. The underlying causes of DM related CNS pathologies are not well understood.

There is a growing body of data indicating that there are cerebral microvascular abnormalities in CNS pathologies such as Alzheimer’s disease, amyotrophic lateral sclerosis (ALS), multiple sclerosis (MS) and stroke^12–15^. Such microvascular abnormalities have not been observed in the case of DM, with the exception of a small scale and preliminary MRI study that found evidence of cerebral microvascular dysfunction in the form of increased blood-brain barrier (BBB) permeability in elderly, non-obese type 2 diabetes patients^16^. CNS vasculature has unique structural and functional characteristics. The metabolic requirements of the brain resemble no other organ, and maintenance of the unique haemostatic environment in the brain is crucial for its function. This environment is maintained by the BBB, which is composed of blood vessels whose endothelial cells are highly specialized. To maintain the brain environment, capillaries isolate the brain from the blood and very tightly control influx/efflux of materials through the capillary wall^17^.

DM animal models studies of BBB dysfunction present contradictory findings and the effect of diabetes on BBB permeability is largely inconsistent in the literature. Early reports noted that diabetes had little or no effect on BBB permeability^18–20^, while more recent studies in animal models of diabetes^21,22^ and MRI evaluation of diabetic patients^16^ present indications for elevated BBB permeability.

The vast majority of studies that examined DM-related BBB dysfunction used the dominant DM rodent model, which is streptozotocin-induced diabetes (STZ). This model is based on administration of a toxin that kills pancreatic beta cells, but also harms the kidney, making it difficult to distinguish effects of diabetes from other systemic drug effects. In addition, the methodology for evaluating BBB dysfunction was based on introducing various tracers into the blood stream followed by clearance (through perfusion) and then trying to extract tracers that penetrate the brain tissue (measurements of tracers in whole brain lysates uses spectrophotometric/radiolabel counts). This methodology is widely used to compare leakage degree between diabetic and control animals. It is very sensitive but lacks spatial resolution and cannot distinguish between different routes of leakage. Some studies use serum constituents such as albumin and IgG, which do not normally cross the BBB, stain with antibodies and image brain sections with microscopy.

In light of the controversy regarding BBB function arising in part from the use of the STZ model, we decided to examine the integrity of the neuro-vascular-unit (NVU) and the permeability of the BBB in the Lepr^db/db^ genetic mouse model of obesity-related type 2 diabetes. Mice homozygous for the mutation in Leptin receptor, do not sense satiety and develop sever hyperphagia leading to morbid obesity, chronic hyperglycemia, pancreatic beta cell atrophy and become hypoinsulinemic.

In contrast to a pharmacological model, using a genetic mouse model enables us to probe different stages along the progression of the diabetic state: pre-hyperglycemia, short-term hyperglycemia and long-term hyperglycemia. Testing BBB function in the Lepr^db/db^ mouse model is especially relevant, as recent studies have shown that these mice also display diabetic-related CNS pathologies^23–26^.

We conducted a thorough examination of barrier permeability to various tracers introduced into the blood stream by both direct confocal-microscopy imaging of BBB capillary in tissue sections and by spectrophotometric measurements of whole brain lysates. Finally, we examined NVU cellular composition and the integrity of the NVU ultrastructure with electron microscopy.

Based on our findings, we suggest that hyperglycaemia is most likely not the underlying cause of previously reported DM-related increased BBB permeability. Furthermore, we propose that BBB hyper-permeability might be an early and transient phenomenon in the Lepr^db/db^ genetic mouse model while stress-related endothelial pathologies do correlate with a short-term diabetic state.

## Results

### BBB permeability of diabetic Lepr^db/db^ mice following short-term hyperglycaemia

The Lepr^db/db^ mouse strain is an established genetic mouse model of type 2 diabetes, extensively used to study the metabolic pathology of obesity-induced insulin resistance leading to hyperglycaemia. Several recent studies used this model to evaluate changes in cerebral vasculature function and found oxidative DNA damage and changes in endothelial glycocalyx^27^, abnormal transport of amyloid-beta^28^ and even leakage of albumin in periventricular regions^29^.

**Figure 1:**
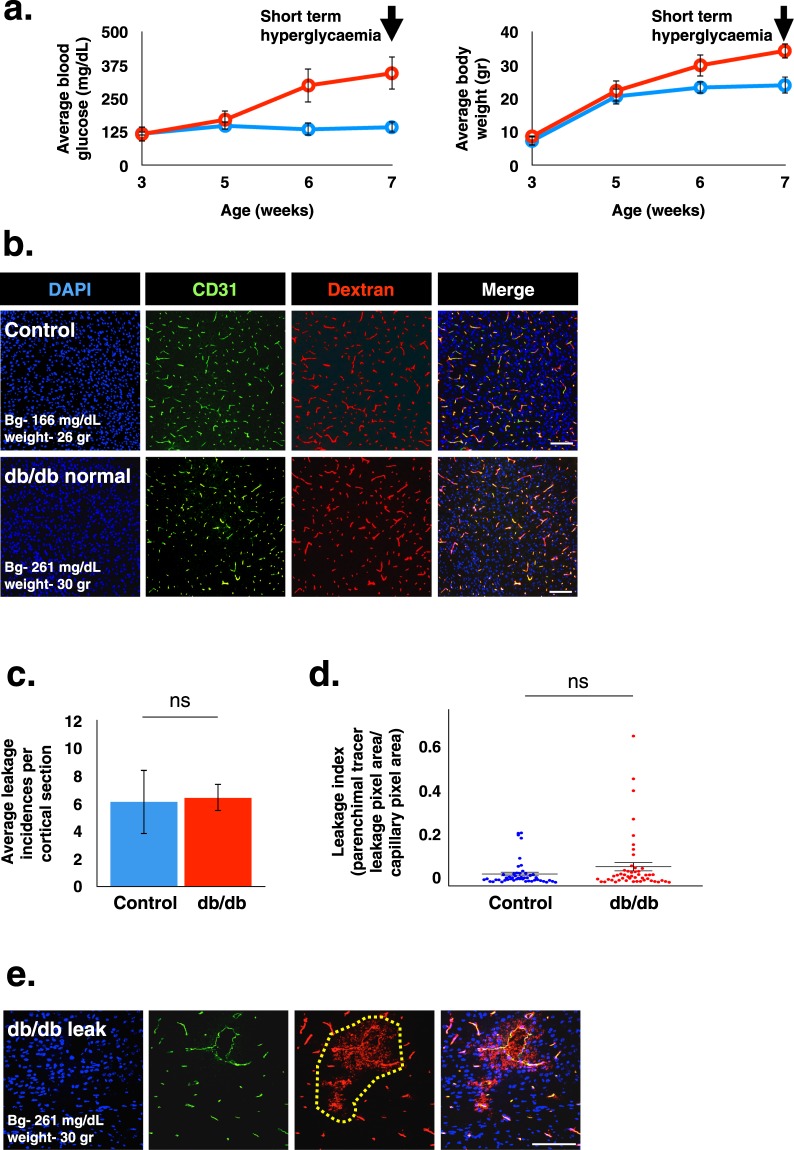
BBB permeability evaluation in Lepr^db/db^ mice following short-term hyperglycaemia. (**a**) Changes in blood glucose levels (left) and body weight (right) of Lepr^db/db^ and control mice. (**b)** Representative images of cortical coronal sections from 10 kDa dextran challenges showing the overall view of normal functioning vessels, both in the diabetic and the control groups. (**c**,**d)** Leakage quantification shows average leakage incidence (**c**) and distribution of leakage areas (**d**) of 10 kDa dextran in 8–10 week-old diabetic Lepr^db/db^ and control mice (ns – non significant, c − P = 0.954, d − P = 0.108 unpaired two-tailed Student’s *t*-test, all data are mean ± s.e.m). (**e)** Examples of 10 kDa dextran extravasations in cortical coronal sections from vessels of 8 week-old diabetic Lepr^db/db^ mice. Most of the leakage incidents were small and infrequent. The image demonstrates examples of the most severe leakage incidents. Scale bar 100 μm. Bg - blood glucose. n = 5 mice for each group (d – n = 49 leakage incidents per group).

We conducted a thorough examination of barrier permeability by introducing various tracers into the blood stream of Lepr^db/db^ mice and controls, starting with testing BBB permeability in short-term hyperglycaemia. Lepr^db/db^ mice started to exhibit elevation of blood glucose levels at five weeks of age and by eight to ten weeks experienced 2–3 weeks of hyperglycaemia and significant elevation in body weight (Fig. 1a).

The majority of previously published studies compare the extent of leakage between diabetic and control animals using measurements of tracers in whole brain lysates. While this is a very sensitive method, it lacks spatial resolution and therefore we chose to first use a direct imaging approach. We used acute challenges (5–10 min) of medium size (10 kDa dextran) and small size (443 Da biotin) tracers, followed by direct tracer imaging of BBB vessels in brain sections with confocal microscopy. Quantification focused on the cortex to avoid regions with higher degrees of normal permeability such as the circumventricular organs (Fig. 1). The overall permeability function of the vascular network seemed not to be grossly affected by the hyperglycaemic state (Figs 1b and S1). For both medium size (Fig. 1e) and small size (Supplementary Fig. S1) tracer challenges, only minor restricted incidents of leakage were noticed demonstrated by tracer fluorescent signal in the brain parenchyma and outside vessels (stained with either CD31 or Glut1). Leakage incidents were restricted and infrequent (1–6 incidents per cortical section in average), but more importantly the trend of leakage incidence being larger in diabetic Lepr^db/db^ mice compared to control normoglycaemic littermates did not reach statistical significance (Figs 1c and S1). Most of leakage areas were small, few capillaries within the vascular network were affected and there was no significant difference in leakage areas between genotypes (Fig. 1d). We concluded that at the short-term diabetic stage and with our methodological approach we could not find evidence for hyperglycaemic-induced BBB permeability.

### BBB permeability of diabetic Lepr^db/db^ mice determined by whole brain lysate spectrophotometric approach

Thus far we failed to demonstrate significant BBB hyper-permeability induced by short-term hyperglycaemia in the Lepr^db/db^ genetic model using a direct imaging approach to assess permeability. Previous studies of the STZ model used measurements of tracer leakage in whole brain lysate^21,22,30,31^, which is considered more sensitive than the direct imaging method since leakage readouts sum signals from whole brains instead of only sampling sections. Moreover, spectrometry (absorbance or fluorescence) reads signals with higher sensitivity than microscopy. In addition, with this method we use a longer tracer circulating time allowing more time for tracer to accumulate in brain tissue if permeability is indeed increased.

**Figure 2:**
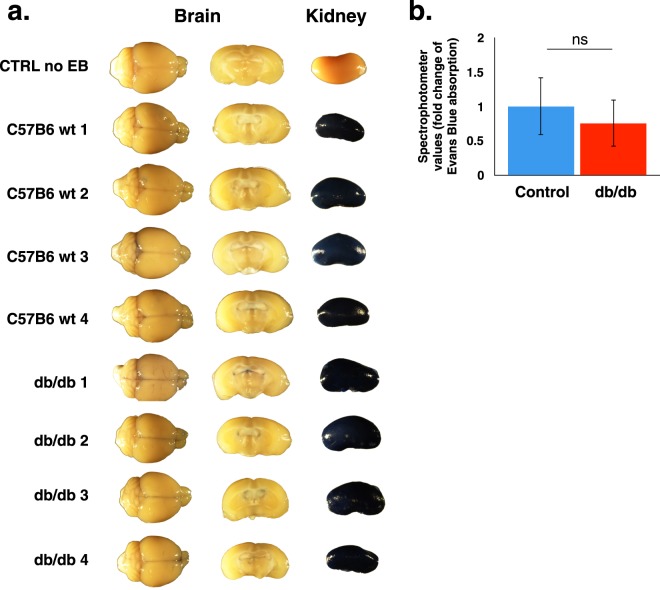
BBB permeability evaluation of diabetic Lepr^db/db^ and control mice, determined by whole brain lysate spectrophotometric measurements of Evans blue (EB) dye. (**a**) Representative images of whole brains, brain coronal views and kidneys, following EB challenges. (**b**) Quantification of EB extravasation by spectrophotometric measurements of whole brain lysates. 8–10 weeks-old diabetic Lepr^db/db^ and control littermate mice were tested (n = 4. ns – non significant, P = 0.811 unpaired two-tailed Student’s *t*-test, all data are mean ± s.e.m).

In order to exclude the possibility that we do not have sufficient sensitivity with the direct imaging approach, we tested permeability in whole brain lysates with the spectrophotometric approach in Lepr^db/db^ mice with short-term hyperglycemia (8–10 weeks-old mice). Evans blue tracer was introduced intravenously and following 3 h of circulation, mice were anaesthetised, perfused with saline (containing heparin) to clear intravascular tracer content and various organs were dissected (Fig. 2). As expected, peripheral organs such as the kidney were heavily stained in both Lepr^db/db^ and control mice reflecting a high degree of capillary permeability (Fig. 2a). No noticeable staining was observed in whole brain or coronal views of both Lepr^db/db^ and control mice brains (excluding some occasional pale blue meningeal folds in both genotypes, Fig. 2a). Moreover, no significant difference was observed by evaluation of tracer extravasation into the brain tissue by spectrophotometric measurements of Evans blue extracts from whole brains (Fig. 2b).

We hypothesized that longer exposure to hyperglycaemia might be needed in order to induce BBB dysfunction. Therefore we examined BBB permeability in long-term hyperglycaemia in Lepr^db/db^ mice (5–6 month-old mice with average blood glucose levels higher then 400 mg/dL, Supplementary Fig. S2) using both the direct imaging approach (data not shown), and the spectrophotometric approach (Supplementary Fig. S2) but found no significant BBB hyper-permeability either.

### BBB ultrastructure abnormalities in diabetic Lepr^db/db^ mice following short-term hyperglycaemia

In order to complement our BBB functional study, we evaluated the ultrastructure of the neuro-vascular unit with electron microscopy. The gross morphology of cortical capillaries in Lepr^db/db^ and control mice appeared normal at 8–10 weeks of age. Ultrastructure properties of endothelial cells related to the barrier selectivity such as tight junction morphology and low abundance of transcytotic vesicles were normal. Ultrastructure properties of the NVU such as astrocyte end-feet and pericyte alignment with the endothelium were also normal. NVU cellular composition of Lepr^db/db^ was similar to that of control mice: cortical tissue co-immuno-stained against the endothelial marker CD31 and the astrocytic marker Aquaporin4 showed co-localization of both markers, demonstrating normal vascular coverage of astrocyte end-feet (Supplementary Fig. S3). Cortical tissue co-immuno-stained against the endothelial marker CD31 and the pericyte marker CD13 showed co-localization of both markers, demonstrating normal pericyte vascular coverage (Supplementary Fig. S3).

**Figure 3:**
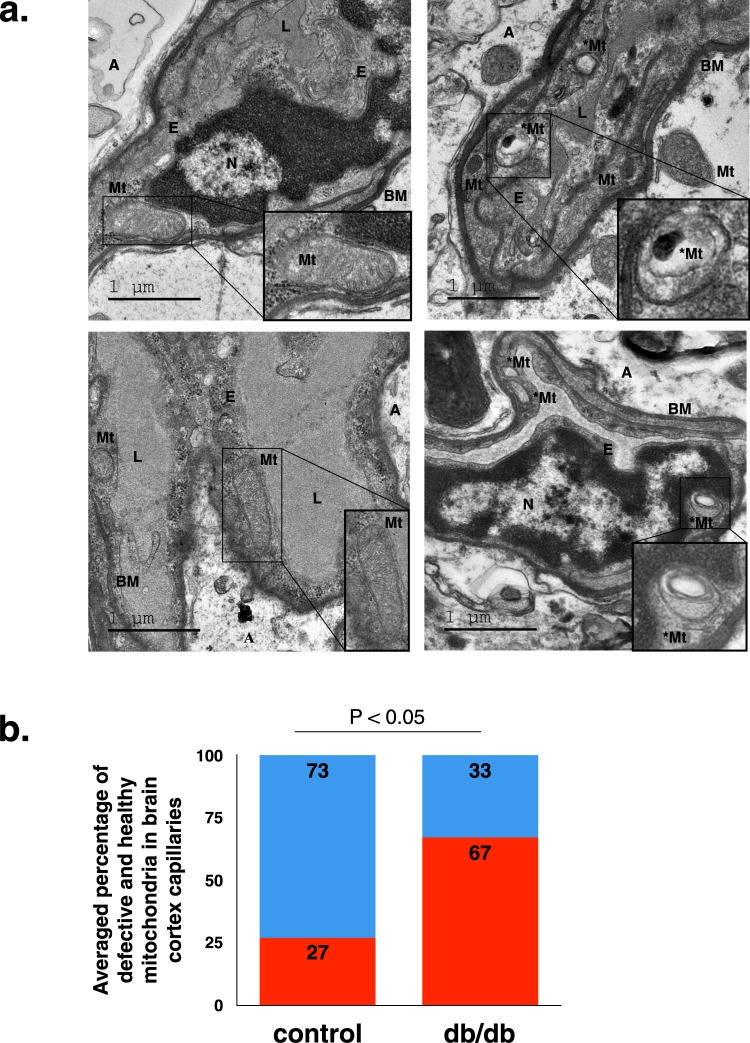
Electron microscopy demonstrating BBB ultrastructural abnormalities in 8 week-old diabetic Lepr^db/db^ cortical capillaries. (**a)** Representative electron micrographs of normal (images in the left column) and abnormal (images in the right column) mitochondrial structure in cortical brain capillaries of 8 week-old diabetic Lepr^db/db^ mice. (**b)** Quantification of defective (red) and healthy (blue) mitochondria in brain cortical capillaries of 8 weeks-old diabetic Lepr^db/db^ and control mice (P < 0.05 unpaired two-tailed Student’s *t*-test). E = endothelium, Mt = normal mitochondrion (with two membranes and mitochondrial crista, *Mt = abnormal mitochondrion (with multiple layers of encapsulating membranes and effacement of mitochondrial crista), BM = basement membrane, A = astrocyte end-foot, N = nucleus. n = 5 mice for each group, n = 6–15 capillaries per mouse.

Nevertheless, electron microscopy imaging of the NVU did reveal some abnormal cellular structures: aberrant mitochondrial structure, blebbed basement membrane and unusual macropinocytotic vesicles at the luminal membrane were all observed (Fig. 3 and Supplementary Fig. S4). While these three ultrastructure abnormalities were more common in Lepr^db/db^ mice, the only abnormality that was significantly and robustly higher in Lepr^db/db^ mice compared to control was mitochondrial structural defects. Quantification of normal and defective mitochondria per capillary profile revealed that the majority of mitochondria in Lepr^db/db^ mice were undergoing some degenerative process, possibly mitophagy as indicated by mitochondrial structures with four layers of encapsulating membranes and effacement of mitochondrial crista (67% of Lepr^db/db^ and 27% in controls, Fig. 3b). To test if this degenerative phenotype had any effect on vascular network properties we measured vascular coverage and network complexity with morphometric tools (vascular labelling in 30 μm tissue sections and analysis of 3D projections, see methods for details). All parameters of vascular network properties tested were unaffected by these mitochondrial abnormalities (Supplementary Fig. S4).

### Leptin receptor deficiency induces early and hyperglycaemia-independent BBB hyper-permeability

**Figure 4:**
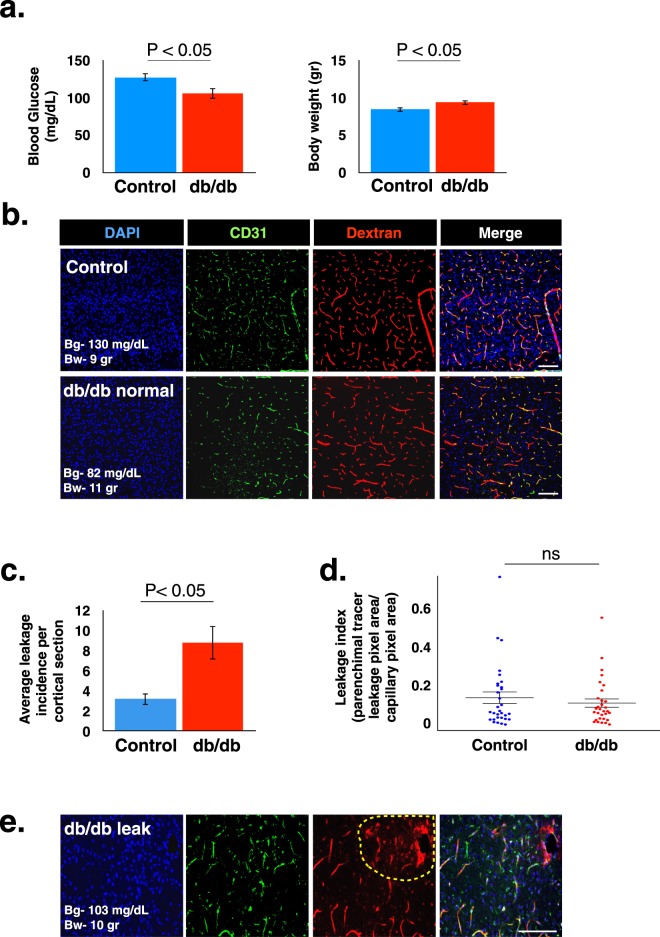
Evaluation of BBB permeability of 3 week-old normoglycemic Lepr^db/db^ and control mice. (**a)** Average blood glucose levels (left) and average body weight (right) of Lepr^db/db^ and control mice. (**b)** Representative images of cortical coronal sections from 10 kDa dextran challenges showing the overall view of normal functioning vessels, both in the diabetic and the control groups. (**c**,**d)** Leakage quantification shows average leakage incidence (**c**) and distribution of leakage areas (**d**) of 10 kDa dextran in 3 week-old normoglycemic Lepr^db/db^ and control mice (a,c – P < 0.05, ns – non significant, d – P = 0.447 unpaired two-tailed Student’s *t*-test, all data are mean ± s.e.m). (**e)** Examples of 10 kDa dextran extravasations in cortical coronal sections from vessels of 3 week-old normoglycemic Lepr^db/db^ mice. The image demonstrates examples of the most severe leakage incidents. Scale bar 100 μm. Bg - blood glucose, Bw - Body weight. n = 5 mice for each group (d − n = 31 leakage incidents per group).

One of the advantages of the genetic over the pharmacological model is the ability to examine mice with the diabetic genotype before they start to enter the hyperglycaemic stage. In our attempts to test diabetic-related BBB hyper-permeability along the progression of the diabetic state, we were surprised to find more frequent leakage incidents in normoglycemic Lepr^db/db^ mice compared to control mice (3 weeks-old mice, Fig. 4). At this stage blood glucose levels in Lepr^db/db^ mice were slightly lower than in controls while body weight was already increased (Figs 4a and S5). Overall leakage incidents were more frequent than in short-term hyperglycaemic mice (Fig. 1c). Interestingly, hyper-permeability was restricted to medium size tracer (10 kDa dextran, Fig. 4c) as small size tracer challenges resulted in non-significant leakage incidence (443 Da biotin, Supplementary Fig. S6). We concluded that in this mouse strain the only significant changes in BBB permeability are apparent approximately two weeks before elevation of blood glucose levels is noticed (Fig. 1a).

## Discussion

Underlying causes of DM-related CNS pathologies are not well understood. Based on epidemiological association between DM and Alzheimer’s disease and on the increasing evidence for involvement of BBB-dysfunction in various neuro-degenerative disorders (including Alzheimer’s disease), it is tempting to speculate a possible contribution of BBB-dysfunction to DM-related CNS pathologies. Nevertheless, interpretations of data concerning BBB-dysfunction from mouse models and DM patients are controversial.

We conducted a thorough examination of barrier permeability using variable tracer challenges to the diabetic BBB of Lepr^db/db^ mice and found no evidence for significant abnormal BBB leakage during the diabetic hyperglycemic state. Our findings are further substantiated by similar results from examination of the non-obese Akita diabetic mouse model (study by Mäe and Genové)^32^. Specifically, BBB permeability of diabetic Lepr^db/db^ mice following short-term or long-term hyperglycaemia was unaltered. This was demonstrated by direct imaging, whole brain lysates spectrophotometric and EM ultrastructure approaches. BBB was evaluated with tracers of variable, molecular size and compositions; 10 KDa dextran, 443 Da biotin and Evans blue - reflecting 70 KDa albumin.

The majority of studies that examined DM-related BBB dysfunction did not use genetic mouse models. An exception are two recent studies that reported abnormal 376 Da sodium-fluorescein tracer leakage in hyperglycemic Lepr^db/db^ mice into the hippocampal neuropil with direct imaging^33^ or in whole brain lysates with spectrophotometric measurements^34^. In our study, we did not detect such a localized abnormality with small size tracers (i.e. biotin) but cannot exclude the possibility that the discrepancy stems from the use of different tracers.

Reports of BBB dysfunction, which resulted in hyper-permeability in animal models, were often describing complex pathological states. Pathologies were not restricted to hyperglycaemia but accompanied by damaged kidney function (STZ), hypertension, ketoacidosis and more^35,36^. Similarly, it is difficult to isolate hyperglycaemia’s contribution in MRI imaging studies of diabetic patients reporting BBB dysfunction. These often involve aged individuals and essentially include subjects with well-controlled type 2 diabetes to intentionally avoid possible confounding attribution to previous hypoglycaemic episodes^16^. Paediatric diabetes MRI imaging studies are usually in Type 1 diabetes patients and might involve ketoacidosis^37^.

In our study we made two interesting new discoveries:

First, we were surprised to find more frequent BBB leakage incidents in normoglycemic 3 week-old Lepr^db/db^ mice compared to wild-type and heterozygous littermate mice. Significant changes in BBB permeability are hyperglycaemia independent, evident approximately two weeks before elevation of blood glucose levels. These are changes in selectivity, relevant to the medium size tracer (10 kDa dextran) but not small size tracer challenges (443 Da biotin). Size-dependency of abnormal BBB permeability might be explained by normal function of endothelial tight junctions (restricting small size molecules) together with abnormal elevated levels of transcytosis (a pathway known to mediate also transport of larger molecules). In future studies it would be interesting to explore the cellular pathway mediating the described BBB leakage. As a preliminary step we analysed mRNA levels of four cellular junction genes (Claudin5, JAM2, ZO1 and Occludin) but found no evidence for differences between Lepr^db/db^ mice and control mice (Supplementary Fig. S7). This finding is in line with a recent study that used a similar approach and found increase in Occludin mRNA levels in Lepr^db/db^ mice^34^. In addition we tested possible changes in Claudin5 expression and localization with immunostaining and did not find differences between Lepr^db/db^ mice and control mice (Supplementary Fig. S7).

Size selective abnormal extravasation of serum substances was previously reported in short-term diabetic STZ rats, through direct imaging demonstrating extravasation of 70 kDa albumin but not 150 kDa IgG nor 170 kDa Complement C3 (rats were also ketoacidotic)^36^. Another study reporting BBB leakage in STZ mice found that leakage was restricted to specific brain regions (significant hyperglycemia-induced BBB disruption was found in cortex, thalamus and midbrain but not the striatum, hypothalamus or hippocampus)^38^. Our data of elevated BBB leakage in normoglycemic 3 week-old Lepr^db/db^ mice was also relevant to the cortex but not to the striatum (Supplementary Fig. S7).

In addition to changes in selectivity, at this age we find a very small but significant decrease in vascular network properties, a decrease not apparent in later stages (Supplementary Fig. S6). This might be related to increased permeability, but also might reflect compensation of possible, unrecognized vascular developmental retardation in this mouse strain. With this regard, as the BBB becomes functional as early as embryonic day 15 (E15), in future studies it would be interesting to evaluate BBB functionality in earlier stages including embryonic development. Since the hyper-permeability we described seems to be corrected at later stages, our current data might reflect retarded barrier formation/maturation related to leptin receptor deficiency.

Second, we found BBB ultrastructure abnormalities in diabetic Lepr^db/db^ mice following short-term hyperglycaemia, of which the most prominent changes were in mitochondrial structure. We suggest that a degenerative process, possibly mitophagy, might represent a cellular response to hyperglycaemia, and might contribute to an endothelial stressed oxidative state. This hypothesis is in line with the endothelial oxidative DNA damage reported in these mice^27^. The observed degenerative mitochondrial process was unexpected, as it was not accompanied by barrier leakage. It is yet to be determined if this mitochondria ultra-structural abnormality reflects mitochondrial dysfunction and what could be its consequences.

Interestingly, DM related degenerative mitochondrial process in the endothelium of various capillary beds is well documented. In the case of diabetes kidney disease, endothelial mitochondrial dysfunction is an early and detrimental step in the pathogenesis^39,40^. Similar ultrastructure or functional changes were reported in corneal endothelium form DM patients^41^, in DM-neuropathy^42^, and in coronary microvasculature^43^. Our findings shows that brain endothelium seems to be more resilient and differs from endothelium of other capillary beds in which the mitochondrial damage is followed by oxidative stress, endothelial cell death and eventual tissue damage.

Based on our findings we suggest that future DM studies shift attention from BBB permeability properties and focus on endothelial metabolic homeostasis and on factors other then hyperglycaemia that might contribute to DM related BBB dysfunction.

## Materials and Methods

### Mice

Lepr^db/db^ mice (purchased from Envigo) and Lepr^db/+^ or Lepr^+/+^ control littermates were used at the age of 3 weeks, 8–10 weeks and 5–6 months. Lepr^db/db^ mice were genotyped by PCR using the following primers: 5′-AGA ACG GAC ACT CTT TGA AGT CTC-3′, 5′-CAT TCA AAC CAT AGT TTA GGT TTG TGT-3′. PCR products were restricted with RsaI restriction enzyme (NEB, cat no. R0167). DNA fragments were separated on 3% agarose gel. All mice were normoglycemic at 3 weeks. Only hyperglycemic (blood glucose >250 mg/dl) Lepr^db/db^ mice were used for the experiments at 8–10 weeks and 5–6 months. Measurements of blood glucose levels were performed with Accu-Chek Performa Test Strips. For all experiments mice were used in a randomized manner, controls were always littermates grown together with mutant mice. For experiments with 3 weeks-old age mice: nine males and eleven females were used. For experiments with 8–10 weeks-old age mice: eleven males and nine females were used. For experiments with 5–6 months-old age mice: twelve males were used. Mice were bred and maintained in the animal facility of the Hebrew University under specific pathogen-free conditions. All animals were treated according to institutional guidelines approved by the Institutional Animal Care and Use Committee (IACUC) at the Hebrew University (Ethics Committee - research number: MD-15-14449-4, NIH approval number: OPRR-A01-5011). All experiments have been reported in compliance with the ARRIVE guidelines for how to report animal experiments, in addition to the guidelines provided in the manuscript.

### Immunostaining

Brain and Kidney tissues were fixed for 24 h at 4 °C in cold 4% paraformaldehyde, incubated for 48 h in 30% sucrose, and embedded in TissueTek OCT (Sakura) on dry ice. 15 μm frozen sections were blocked with 10% normal horse serum (NHS)/10% BSA/0.5% triton X-100 solution for 1 h at RT, and incubated in primary antibody diluted in blocking solution overnight at 4 °C. Sections were washed in PBS and incubated with fluorophore-conjugated secondary antibodies. Sections were subsequently coverslipped with fluorescent mounting medium supplemented with DAPI nuclear counterstain (Southern Biotech Dapi Fluoromount-G cat no. 0100-20).

For sulfo-NSH-biotin tracer experiments, 100 μm coronal frozen sections from the same brain regions were placed in 24-well plates and washed with PBS and 0.2% Triton-PBS. Tissue was blocked with 10% NHS/10% BSA/0.5% triton X-100 solution overnight at RT, incubated in primary antibody for endothelial vessel marker diluted in blocking solution at RT for several hours and then moved to overnight incubation at 4 °C. Sections were washed in PBS and incubated with streptavidin and fluorophore-conjugated secondary antibodies. Sections were subsequently coverslipped with fluorescent mounting medium supplemented with DAPI nuclear counterstain. All incubations were done while shaking.

The following antibodies were used: hamster anti-mouse CD31 (Bio-Rad cat no. MCA 1370Z, 1:100), rabbit anti-GLUT1 (Millipore cat no. 07-1401, 1:100), and rabbit anti-laminin (Thermo Scientific, cat no. RB 082-A0 1:100), rabbit anti-Aquaporin4 (Millipore cat no. AB3594, 1:400), rabbit anti-CD13 (Cell Signalling Technologies cat no. 32720 1:200), rabbit anti-Claudin5 (Thermo Scientific, cat no. 34–1600 1:100). Secondary antibodies conjugated to CY2 (1:200), CY3, or CY5 (1:500) were from Jackson Immunoresearch. Immunofluorescence images were captured using a Nikon (Nikon eclipse Ni, objective x20 and x40 with Nikon C2 camera and Nis-Elements software) and Zeiss (Zeiss LSM710, objective x20 and x40 with Zen10 software) confocal microscopes.

### Transmission electron microscopy

Brain tissue was fixed with 4% paraformaldehyde and 2.5% glutaraldehyde (Electron Microscopy Sciences [EMS]), post fixed with 1% osmium tetroxide (Sigma-Aldrich), and dehydrated with increasing concentrations of ethanol, followed by propylene oxide (Sigma-Aldrich). For embedment, we used Agar 100 Resin (Agar Scientific). For imaging, we used 80 nm sections stained with 5% uranyl acetate for 10 min, followed by 10 min with lead citrate. Samples were visualized with a JEM-1400 Plus transmission electron microscope (Jeol) equipped with a Gatan CCD camera. For EM quantification, 15 cortical capillaries of each mouse, randomly selected, were analysed using the ImageJ software.

### BBB Integrity

An *in vivo* permeability assay was performed using three different molecular size tracers: 65,000 Da Evans blue, 10,000 Da dextran-tetramethylrhodamine and 443 Da sulfo-NHZ-biotin.

#### For dextran-tetramethylrhodamine leakage evaluation

deeply anesthetized mice were injected with dextran-tetramethylrhodamine (Thermo Fisher Scientific, cat no. D1817) 2 mg/20 g body weight to the left ventricle and it was allowed to circulate in the blood stream for 5–10 min. After perfusion, brain was dissected, fixed and treated as described for immunohistochemistry.

#### For sulfo-NHS-biotin leakage evaluation

deeply anesthetized mice were transcardially perfused through the left ventricle (mini pump II, Harvard apparatus) with 20 ml/30 g body weight of 0.5 mg/ml sulfo-NHS-biotin (Thermo Scientific, cat no. 21217) in PBS. After perfusion, the brain was dissected, fixed and treated as described for immunohistochemistry.

#### For quantification of Evans blue extravasation

BBB permeability was evaluated by measuring extravasation of Evans blue (EB) dye (Sigma cat no. E-2129), which can bind to serum albumin after intravenous injection and, therefore, has been used as a tracer for serum albumin. EB dye (2% in saline, 4 mL/kg) was injected trough the tail vein and allowed to circulate in the blood stream for 3 h. Deeply anesthetized mice were perfused with cold phosphate-buffered saline including for 15 min via the left ventricle to remove the intravascular dye. After perfusion, brain and kidney were dissected. The excised tissues were weighed, minced and homogenized in 2.5 ml of PBS and mixed with 2.5 ml of 60% trichloroacetic acid. Samples were cooled for 30 min followed by centrifugation (4 °C, 1000 *g*, 30 min) to precipitate the tissue. The supernatant was subjected to measurement of the absorbance of EB at 610 nm using a spectrophotometer. EB dye was expressed as micrograms per milligram of brain tissue against a standard curve. For macroscopic evaluation, after perfusion with ice-cold PBS, the brain was rapidly removed and coronally sectioned at 1 mm thickness and photographed.

### Quantification of BBB leakage

All quantifications were done by a person blind to the genotype in a randomized manner.

#### For dextran-tetramethylrhodamine leakage quantification

four non-serial 15 μm coronal sections from the same location per animal were immunostained for endothelial vessel marker and scanned under the microscope. A leakage incident was defined when a tracer was localized outside the endothelial lumen and valued as 1. Quantification was focused on the cortical and striatum areas. Leakage from other brain areas was excluded. An average was calculated for every four non-serial sections of each mouse and for all mice in each group.

#### Evaluation of leakage areas

Quantification was performed using Angiotool software^44^. 6–10 images per animal were obtained. The same background subtraction and auto threshold parameters were applied to max z-projections of both CD31 (endothelium cell marker), and dextran (tracer) channels. Masks of both channels were generated and the leakage index was defined as the area of parenchymal tracer mask (tracer minus vessel) normalized to the area of the vessels mask.

#### For sulfo-NHS-biotin leakage quantification

two non-serial 100 μm coronal sections from the same location per mouse were immunostained for streptavidin and endothelial vessel marker and scanned under the microscope. A leakage incident was defined when a tracer was localized outside the endothelial area and valued as 1.

Quantification was focused on the cortical and hippocampal areas. Leakage from other brain areas was excluded. Average was calculated for every two non-serial sections of each mouse and for all mice in each group. 3D images from the cortex and hippocampus were taken and processed to max z-projections of both GLUT1/laminin (endothelium vessel markers) and sulfo-NHS-biotin (tracer) channels, using ImageJ software.

### Vessel profiling

30 μm cryo-sections were stained for CD31 and imaged with a confocal microscope (Nikon Eclipse Ni, objective x20 with Nikon C2 camera and Nis-Elements softwareNikon TE-2000, objective x20 with EZ-C1 software). Z-stack images (x-y 30 intervals of 1 μm) were analysed for vessel parameters using Angiotool software^44^. Two group comparisons were analysed using an unpaired two-tailed Student’s t test.

Forebrain tissue of 3 weeks-old Lepr^db/db^ and Lepr^db/+^ mice was carefully cleared of meninges and choroid plexus and total RNA was purified using BIO TRI RNA reagent (bio-lab). Total RNA (2 µg) was used for first-strand cDNA synthesis using random primers and reverse transcriptase (Applied Biosystems). Real-time PCR was performed with SYBR Green master mix (Applied Biosystems) in 96-well plates using the Applied Biosystems real-time thermal cycler 7300. All reactions were performed in triplicates with three-four biological replicates. The relative amount of mRNA was calculated using the comparative CT method after normalization to *Pecam*. Briefly, we calculated ΔCt values between each gene and *Pecam*, and ΔΔCt values were calculated between the ΔCt of each replicate and the average ΔCt for Lepr^db/db^ replicates. The following primers were used:

*Pecam* forward (5′-CTCACGCTGGTGCTCTATG-3′)

and reverse (5′-CCATTCATCACCTCCCATGAT-3′),

*Claudin 5* forward (5′-TGCCGCTCTGCTGGTTC-3′)

and reverse (5′-CAGCTCGTACTTCTGTGACACC-3′),

*Tjp1* forward (5′-CTATATGGGAACAGCACACAGT-3′)

and reverse (5-AATGAGGATTATCTCTTCCACCAG-3′),

*Jam2* forward (5′-GCTGCTACACTACTTGATCGTC-3′)

and reverse (5′-TGGAACTCTATTACTGTGACTTCTTG-3′).

Occludin forward (5′-CCAATGTTGAAGAGTGGGTTAAA-3′).

and reverse (5′-TTTGCCATTGGAGGAGTAGG-3′).

### Statistical analysis

Two group comparisons were analysed using an unpaired two-tailed Student’s t test. Sample size for all experiments was determined empirically using standards generally employed by the field: a minimum of three animals per group in each experiment (most included five animals) and a minimum of four tissue sections of each brain. No data was excluded when performing statistical analysis. Standard error of the mean was calculated for all experiments and displayed as errors bars in graphs. Statistical details for specific experiments can be found in the Figure Legends.

## Supplementary information

Supplementary Figures
