# Supplementary material for: Leptin receptor deficiency induces early, transient and hyperglycaemia-independent blood-brain barrier dysfunction
Source: Sci Rep. 2019 Feb 27;9:2884. doi: 10.1038/s41598-019-39230-1 (PMC6393679; doi:10.1038/s41598-019-39230-1)
Supplement: Supplementary file 1 — Supplementary Figures [file 41598_2019_39230_MOESM1_ESM.pdf]

Leptin receptor deficiency induces early, transient and hyperglycaemia-independent blood-brain barrier dysfunction

Noa Corem, Shira Anzi, Sivan Gelb, Ayal Ben-Zvi*

Department of Developmental Biology and Cancer Research, The Institute for Medical Research Israel-Canada, Faculty of Medicine, Hebrew University of Jerusalem, Jerusalem 91120, Israel

*Corresponding author: Ayal Ben-Zvi PhD, Department of Developmental Biology and Cancer Research, The Institute for Medical Research Israel-Canada, Faculty of Medicine, Hebrew University of Jerusalem, Jerusalem 91120, Israel. Ayalb@ekmd.huji.ac.il, +97226757624

Supplementary Figure 1

Blood glucose and body weight of 8w old Lepr $^{db/db}$ mice - SulfoBiotin tracer challenge experiment

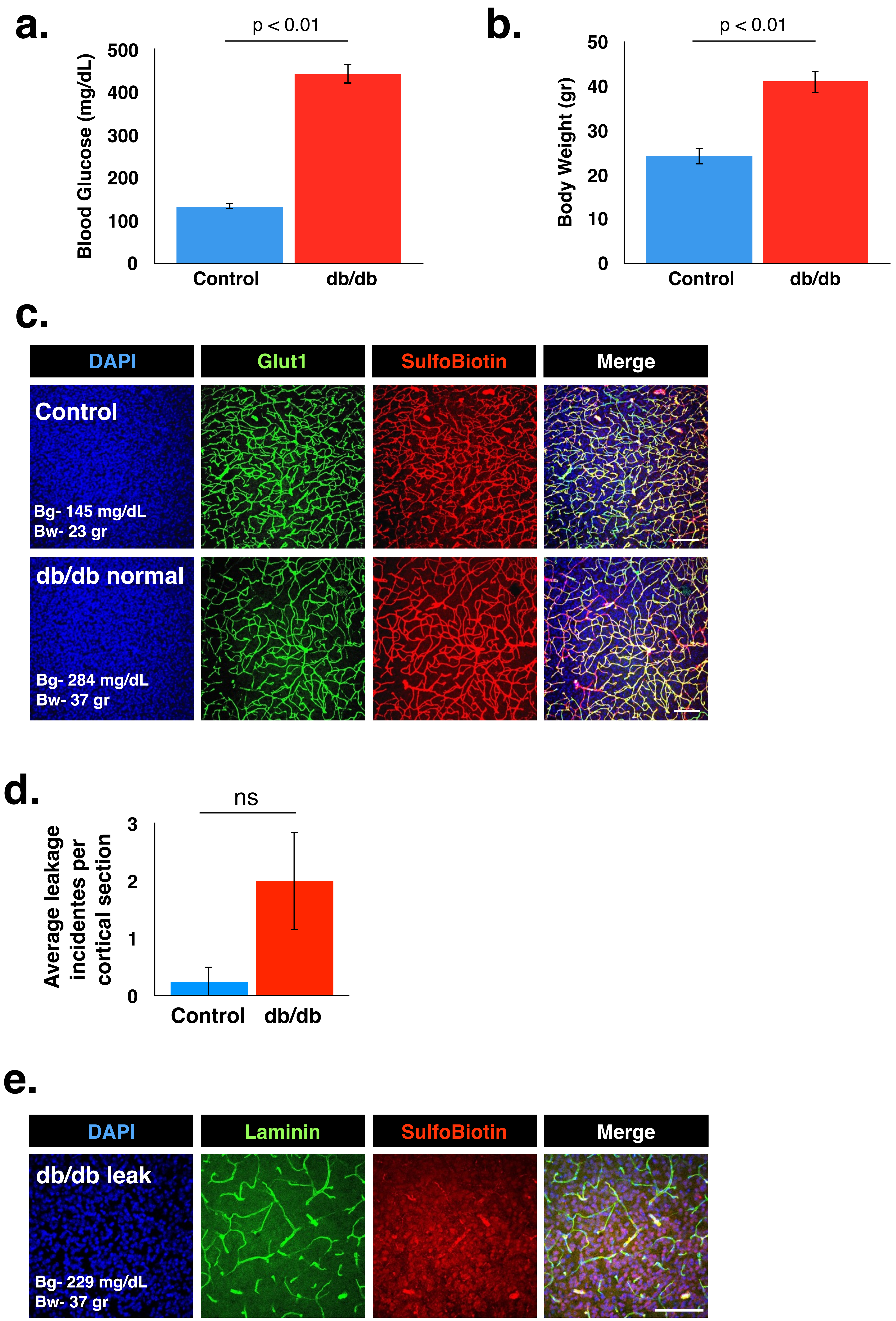

Supplementary Figure 1

Evaluation of BBB permeability to sulfo-biotin in Lepr$^{db/db}$ mice following short-term hyperglycaemia.

a, Average blood glucose levels in 8-10 week-old diabetic Lepr$^{db/db}$ and control mice used for the sulfo-biotin assay. **b**, Average body weight in 8-10 week-old diabetic Lepr$^{db/db}$ and control mice used for the sulfo-biotin assay (**a**,**b** – $P<0.01$, unpaired two-tailed Student's *t*-test, all data are mean ± s.e.m). **c**, Representative images of cortical coronal sections from 443 Da sulfo-biotin challenges showing the overall view of normal functioning vessels, both in the diabetic and the control groups. **d**, Average leakage incidents of 443 Da sulfo-biotin per cortical coronal section of 8-10 week-old diabetic Lepr$^{db/db}$ and control mice ($P=0.121$ unpaired two-tailed Student's t-test). **e**, Examples of 443 Da sulfo-biotin extravasations in cortical coronal sections from vessels of 8-10 week-old diabetic Lepr$^{db/db}$ mice. Most of the leakage incidents were small and infrequent. The image demonstrates examples of the most severe leakage incidents. Scale bar 100 µm. Bg - blood glucose, Bw – Body weight. n=4 mice for each group. All data are mean ± s.e.m.

Supplementary Figure 2

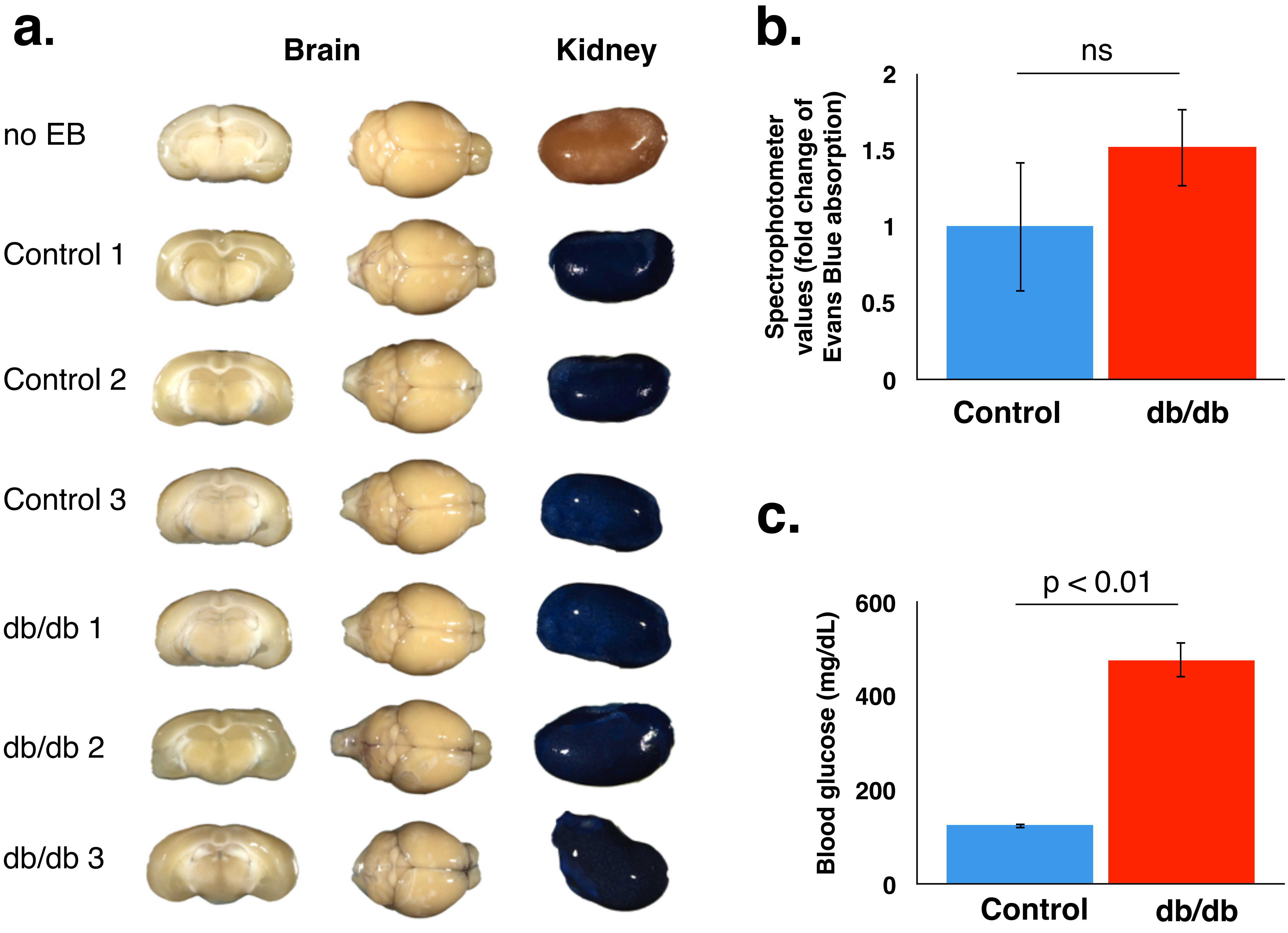

Supplementary Figure 2

BBB permeability evaluation of diabetic Lepr$^{db/db}$ and control mice, determined by whole brain lysate spectrophotometric measurements of Evans blue (EB) dye.

a, Representative images of whole brains, brain coronal views and kidneys, following EB challenges. **b**, Quantification of EB extravasation by spectrophotometric measurements of whole brain lysates. 5-6 months-old diabetic Lepr$^{db/db}$ and control littermate mice were tested. **c**, Average blood glucose levels 5-6 months-old diabetic Lepr$^{db/db}$ and control littermate mice (**b**, ns – non significant, P=0.510, **c**, P<0.01 unpaired two-tailed Student's t-test). n=5 Lepr$^{db/db}$ and 7 control mice. All data are mean ± s.e.m.

Supplementary Figure 3

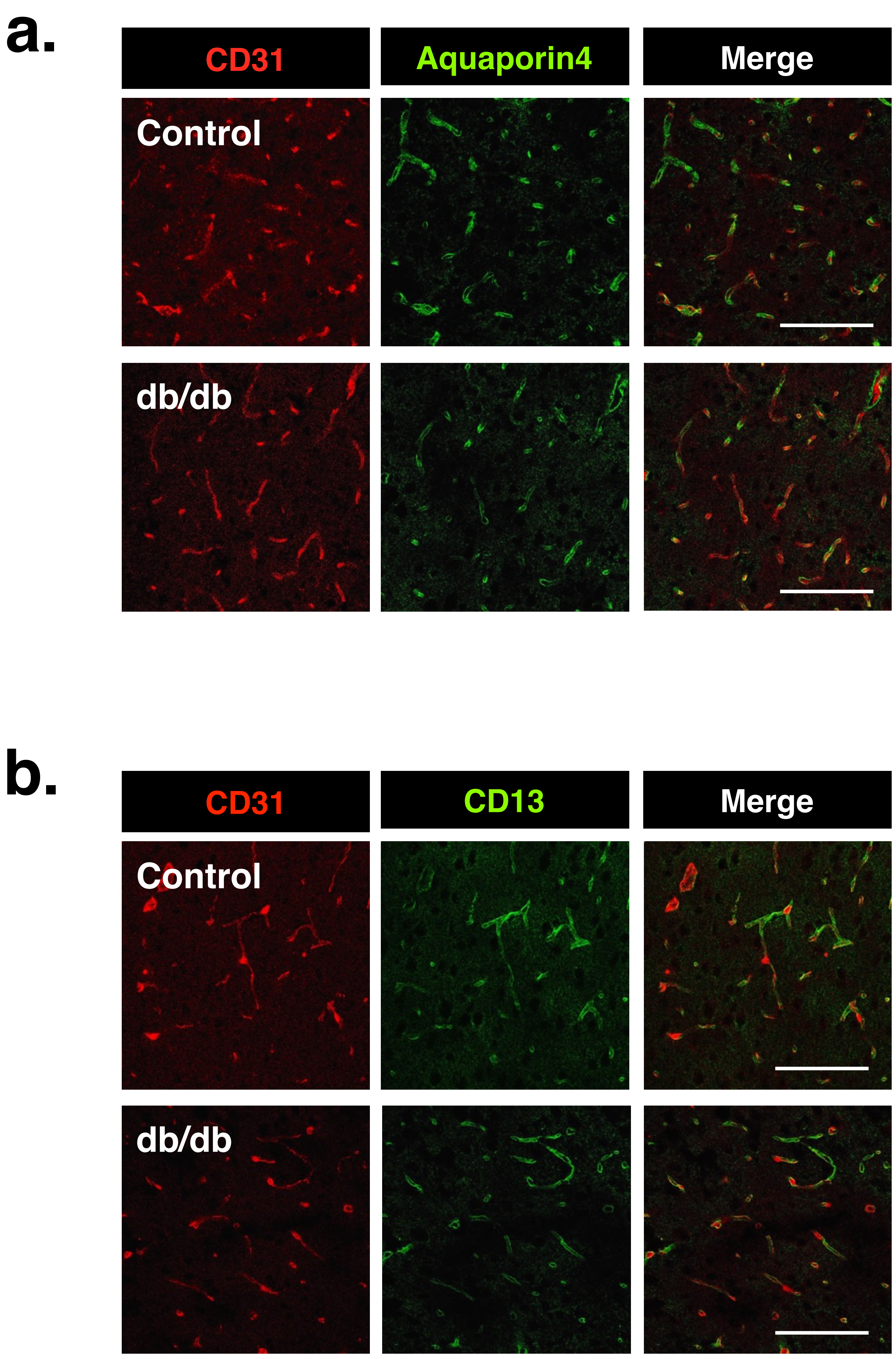

Supplementary Figure 3

NVU cellular composition of Lepr$^{db/db}$ cortex

NVU cellular composition of 8 week-old diabetic Lepr$^{db/db}$ and control mice. **a**, Cortical tissue co-stained with CD31 (Endothelial marker - red) and Aquaporin4 (Astrocyte end-feet marker - green) demonstrating normal vascular coverage of astrocyte end-feet. **b**, Cortical tissue co-stained with CD31 (Endothelial marker - red) and CD13 (Pericyte marker- green) demonstrating normal vascular coverage of pericytes. Scale bar 100 µm. n=3 mice for each group.

Supplementary Figure 4

BBB ultrastructural abnormalities and vasculature topology in 8 weeks-old diabetic Lepr$^{db/db}$ mice.

a. Damaged mitochondria

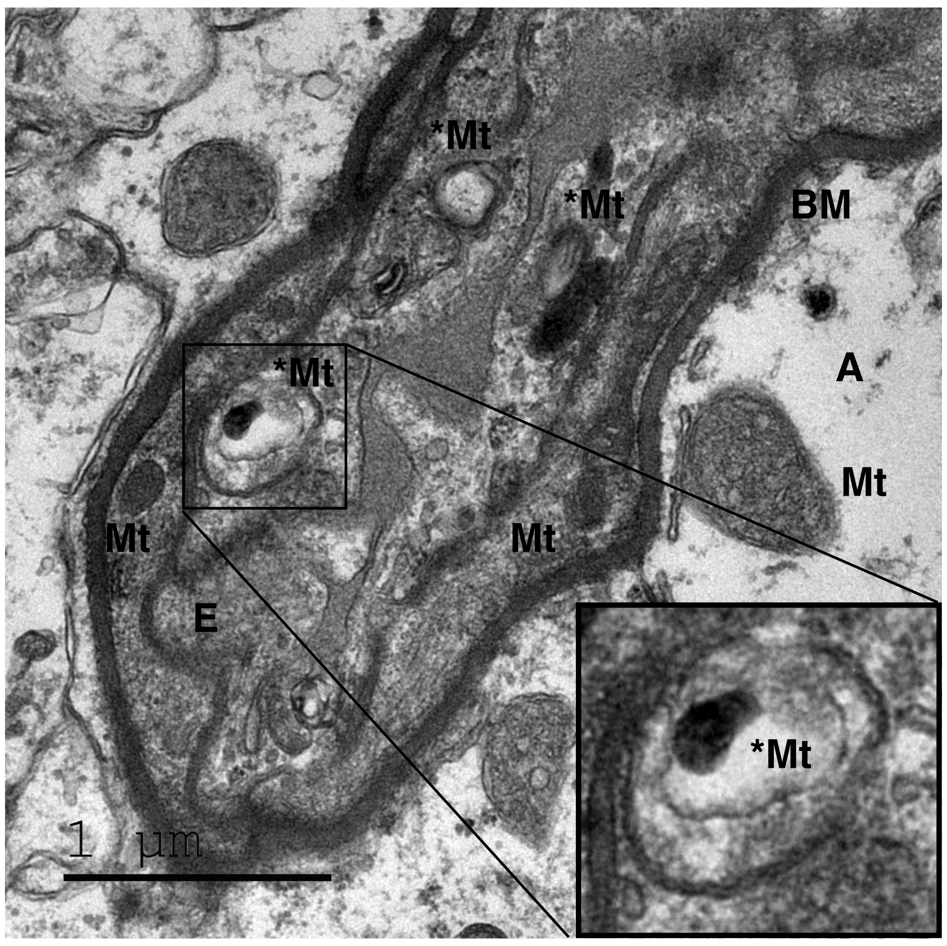

b. Damaged basement membrane

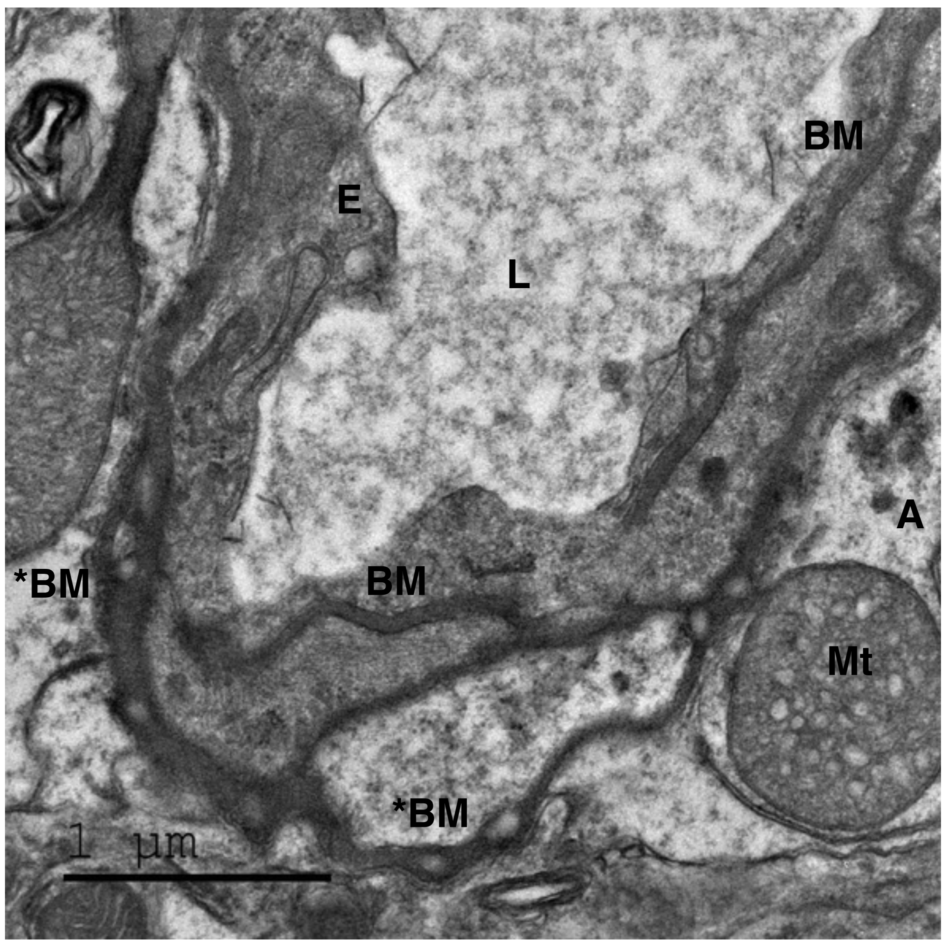

c. Macropinocytosis

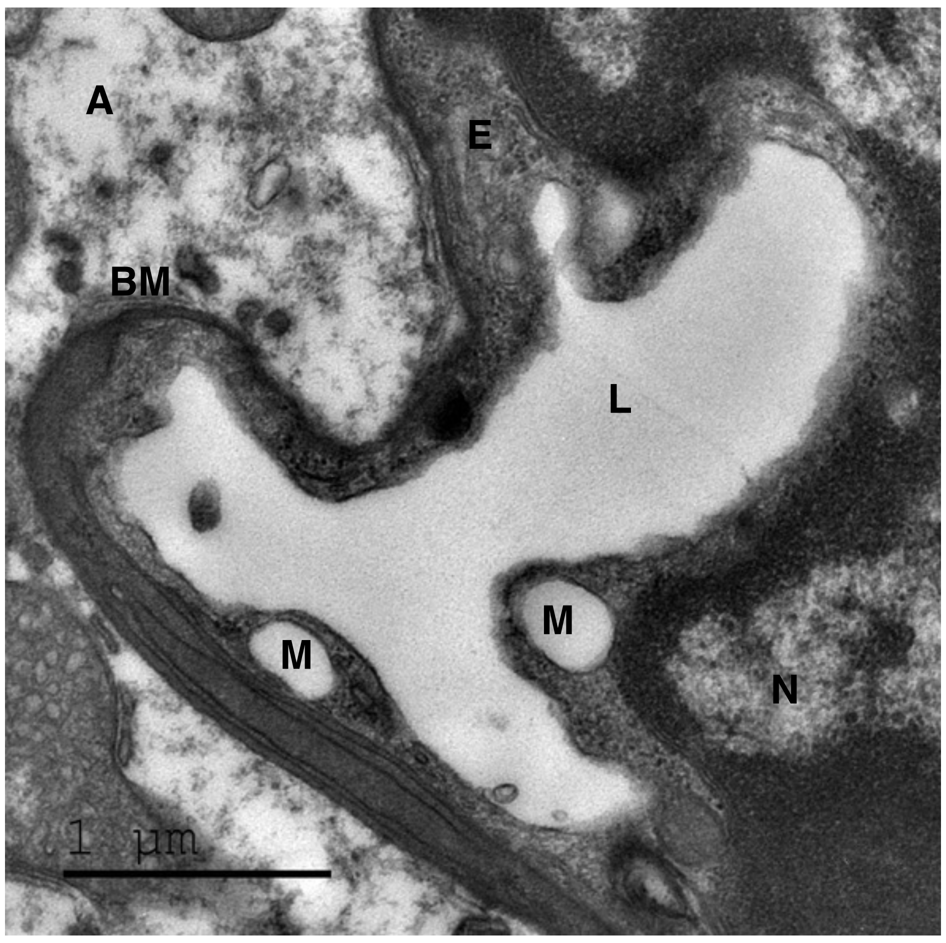

d. **Cortical vasculature topology of 8w old mice**

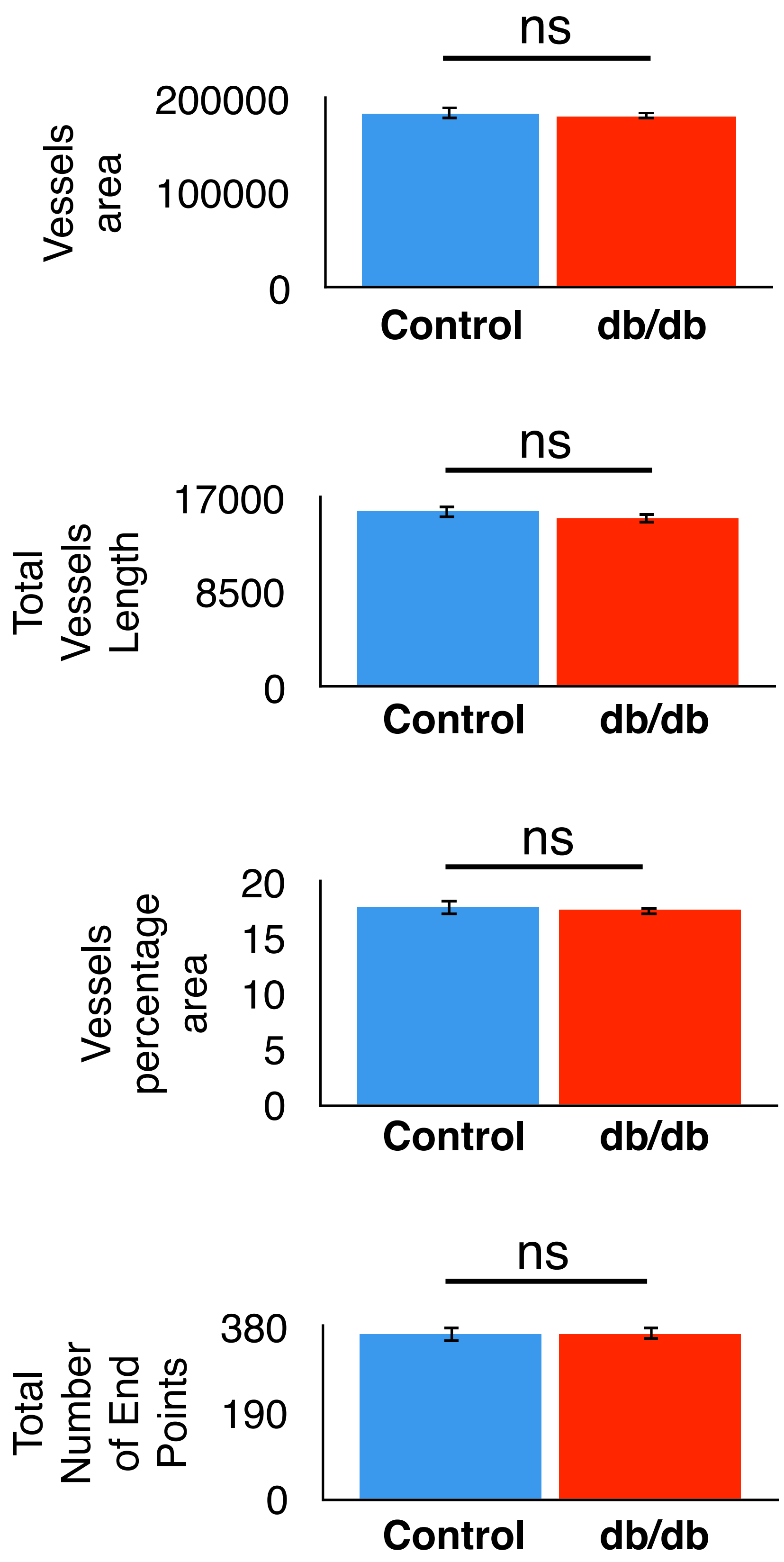

Supplementary Figure 4

BBB ultrastructural abnormalities and vasculature topology in 8 weeks-old diabetic $Lepr^{db/db}$ mice.

a-**c**, Representative electron micrographs of BBB ultrastructural abnormalities in cortical brain capillaries of 8 weeks-old diabetic $Lepr^{db/db}$ mice. **a,** Damaged mitochondria. **b,** Abnormal basement membrane structure appears as a dark line discontinued by clear blebs (*BM) while normal basement membrane appears as a continuous dark line (BM). **c,** Macropinocytosis. E=endothelium, Mt=mitochondrion, *Mt=abnormal mitochondrion, BM=basement membrane, *BM= abnormal basement membrane, A=astrocyte end-foot, L=capillary lumen, N=nucleus, M=macropinocytosis vesicles. **d**, Angio Tool software[44] was used for quantitative analysis of alteration in the blood vessels topology during diabetes development in $Lepr^{db/db}$ mice. No change in vasculature features of hyperglycemic 8 weeks-old $Lepr^{db/db}$ compared with $Lepr^{db/wt}$ and $Lepr^{wt/wt}$ control littermates (vessels area ($p=0.749$), total vessel length ($p=0.381$), vessel percentage area ($p=0.738$), number of points ($p=0.943$). Unpaired two-tailed Student's *t*-test). n=3 mice for each group. All data are mean ± s.e.m.

Supplementary Figure 5

Blood glucose and weight of 3w old mice, Dextrane tracer challenge

Blood glucose and weight of 3w old mice, SulfoBiotin tracer challenge

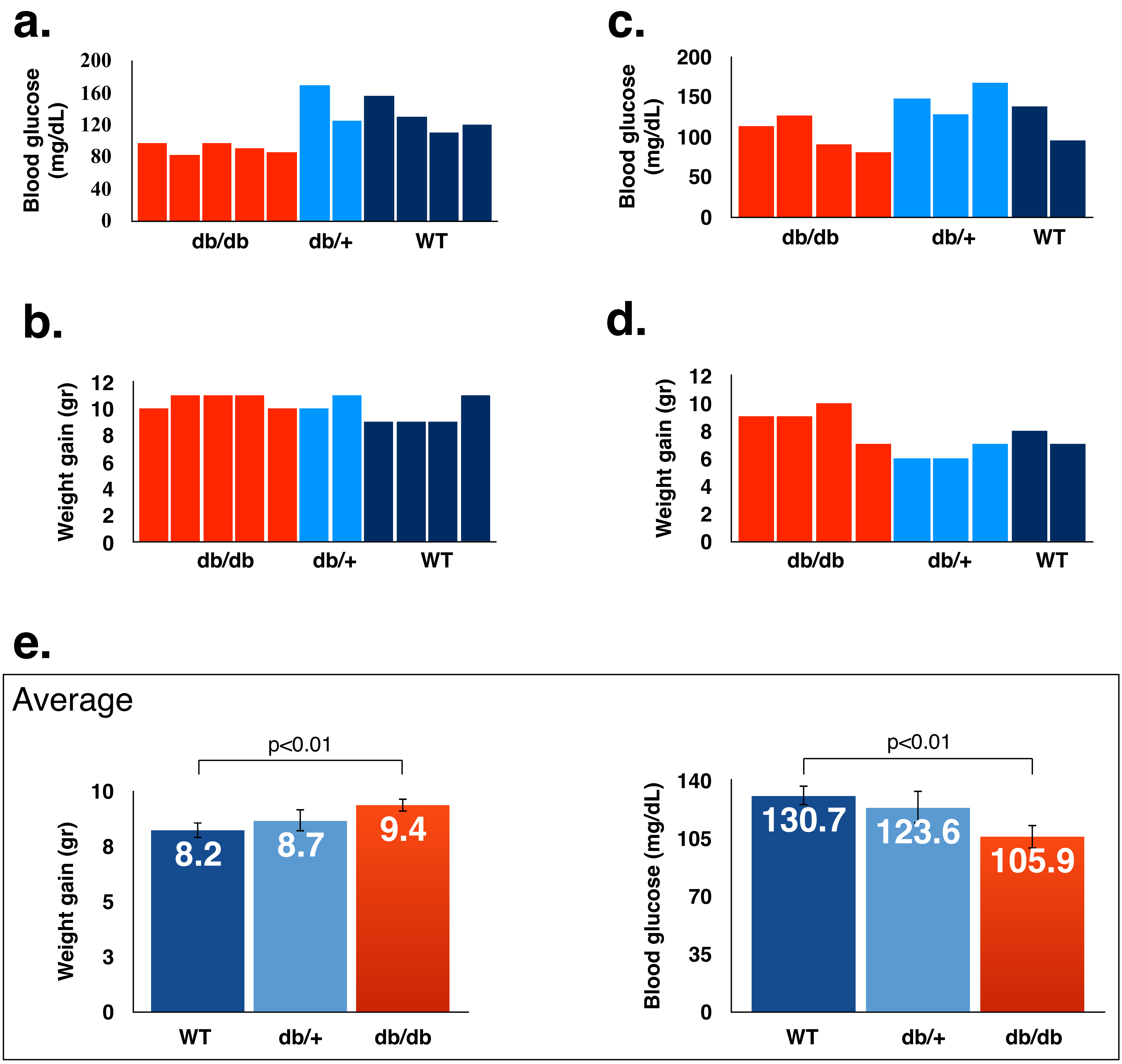

Supplementary Figure 5

Blood glucose and body weight of 3 week-old normoglycemic Lepr$^{db/db}$ and control mice

a,**c**, Blood glucose levels in 3 weeks-old normoglycemic Lepr$^{db/db}$ and control individual mice, used for the dextran permeability assay (**a** - related to Figure 4) and for the sulfo-biotin assay (**c** - related to Figure S6). **b**,**d**, Body weight in 3 week-old normoglycemic Lepr$^{db/db}$ and control individual mice, used for the dextran permeability assay (**b** - related to Figure 4) and for the sulfo-biotin assay (**d** - related to Figure S6). **e**, Averages of blood glucose levels and body weight values of all groups in the 3-week-old mice assays. All data are mean ± s.e.m.

Supplementary Figure 6

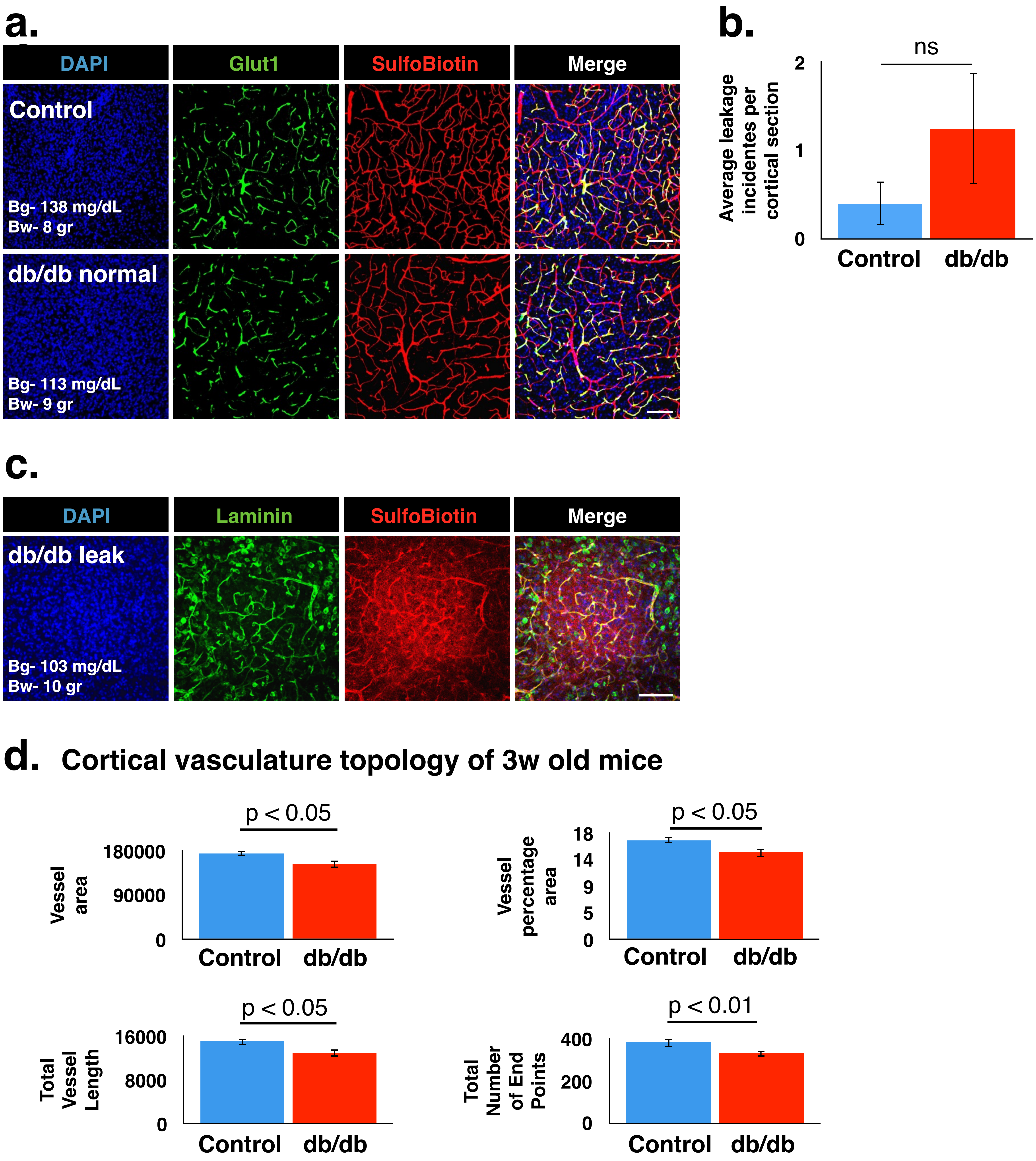

Supplementary Figure 6

Quantitative analysis of alteration in blood brain vessels features and small size tracer challenges in normoglycemic 3 weeks old Lepr$^{db/db}$ mice.

a, Representative images of cortical coronal sections from 443 Da sulfo-biotin challenges showing the overall view of normal functioning vessels, both in the normoglycemic 3 weeks-old Lepr$^{db/db}$ mice and the control groups. **b,** Average leakage incidents of 443 Da sulfo-biotin per cortical coronal section of 8 week-old diabetic Lepr$^{db/db}$ and control mice (ns – non significant , P=0.277 unpaired two-tailed Student's t-test). **c,** Examples of 443 Da sulfo-biotin extravasations in cortical coronal sections from vessels of 8 week-old diabetic Lepr$^{db/db}$ mice. Most of the leakage incidents were small and infrequent. The image demonstrates examples of the most severe leakage incidents. Scale bar 100 µm. Bg - blood glucose, Bw – Body weight. n=4 Lepr$^{db/db}$ and 5 control mice. **d,** Angio Tool software[44] was used for quantitative analysis of alteration in blood vessels topology. Minor but significant reduction in different vasculature features of normoglycemic 3 weeks-old Lepr$^{db/db}$ compared with Lepr$^{db/wt}$ and Lepr$^{wt/wt}$ control littermates (vessels area ($p<0.05$), total vessel length - ($p<0.05$), vessel percentage area ($p<0.05$), number of end points ($p<0.01$). Unpaired two-tailed Student's t-test). n=5 Lepr$^{db/db}$ and 6 control mice. All data are mean ± s.e.m. for each group.

Supplementary Figure 7

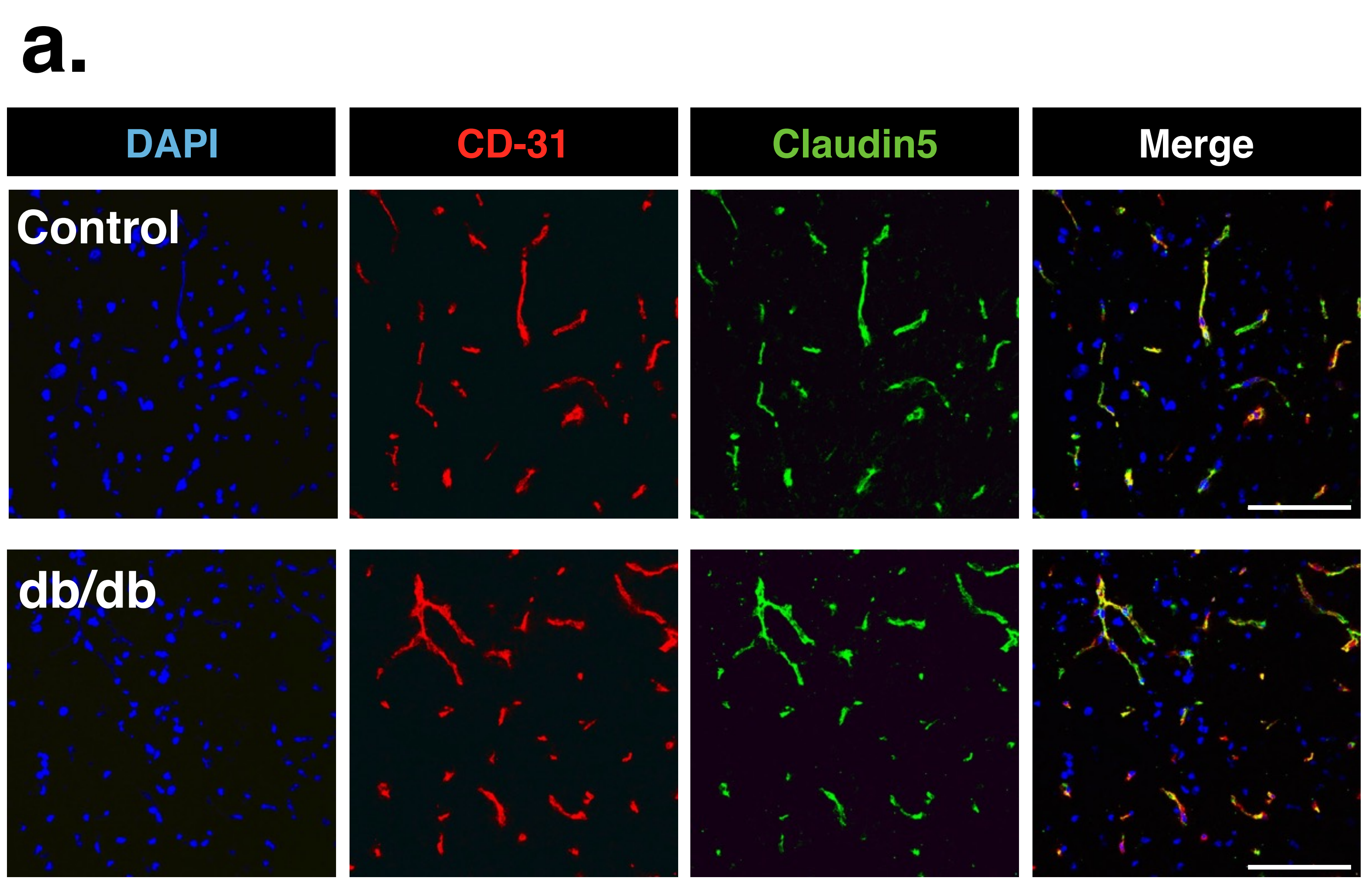

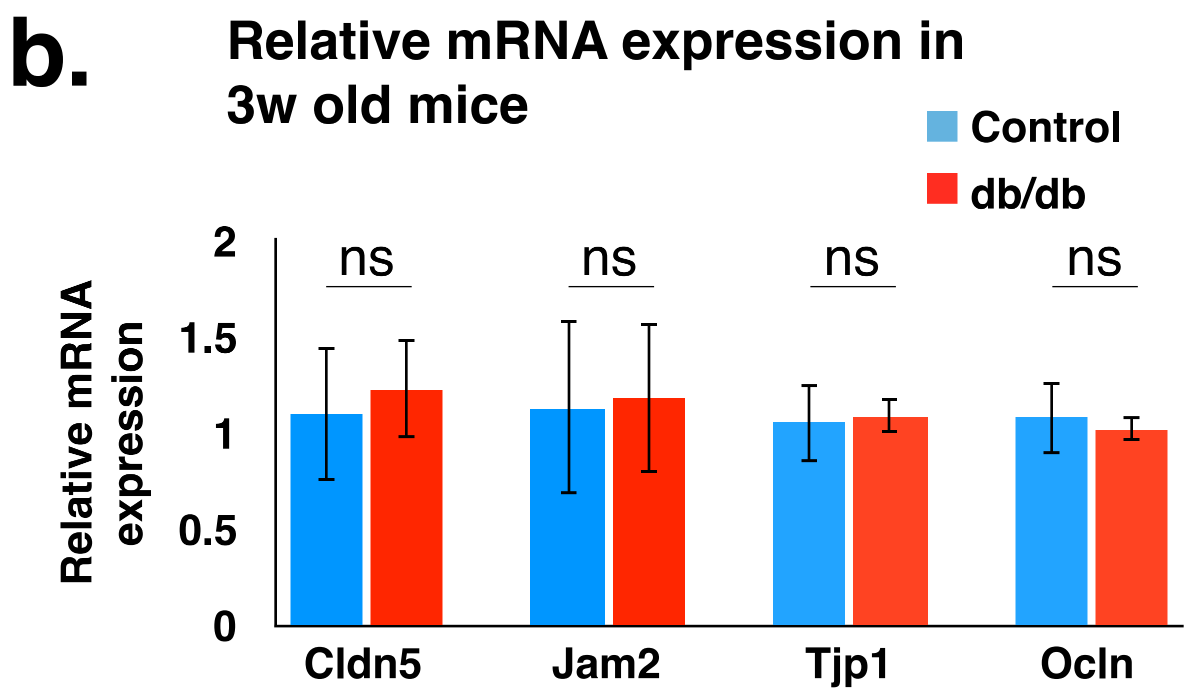

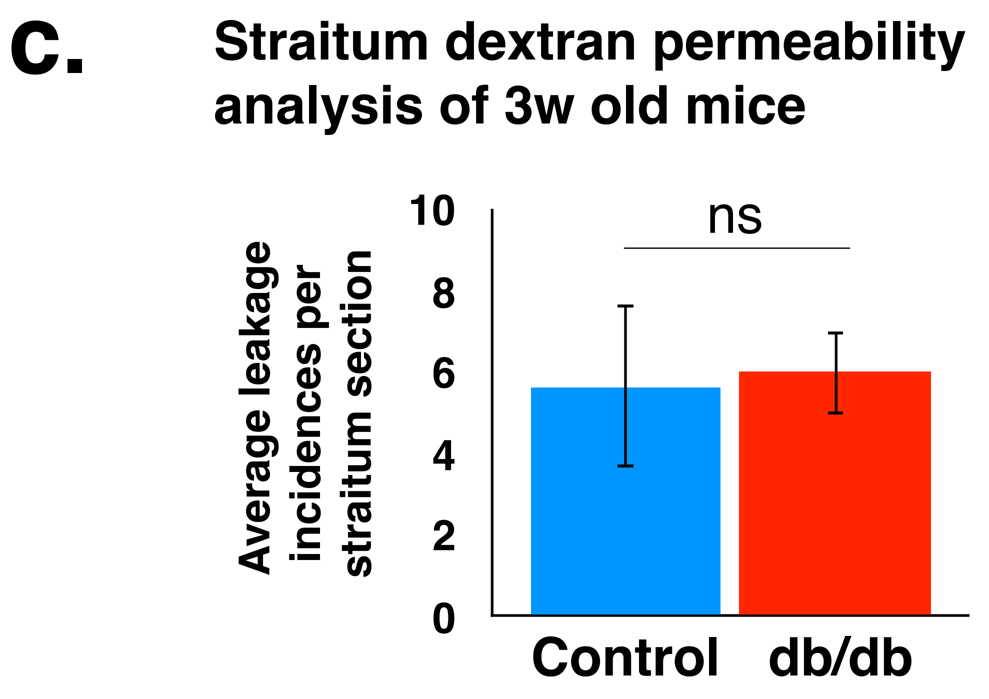

Supplementary Figure 7

Additional analysis of permeability, cellular junctions mRNA levels and immunostaining of normoglycemic 3 weeks-old Lepr$^{db/db}$ and control mice.

Tight junctions mRNA levels and Claudin5 immunostaining of 3 week-old diabetic Lepr$^{db/db}$ and control mice. **a,** Cortical tissue co-stained with CD31 (red) and Claudin5 (green) demonstrating normal Claudin5 endothelial expression and localization. Scale bar 100 µm. n=3 mice for each group. **b,** mRNA levels of four cellular junction genes (Claudin5, Jam2, Tjp1 (ZO1) and Ocln (Occludin1)) was evaluated with real-time PCR (ns – non significant, Claudin5 P=0.581, Jam2 P=0.878 Tjp1 P=0.736, Ocln P=0.599 unpaired two-tailed Student's t-test). n=3 Lepr$^{db/db}$ and 4 control mice. **c,** Leakage quantification shows average leakage incidents of 10 kDa dextran in the striatum of 3 week-old normoglycemic Lepr$^{db/db}$ and control mice (ns – non significant , P=0.892 unpaired two-tailed Student's t-test). n=3 mice for each group. All data are mean ± s.e.m. for each group.
